# Assessment method for new sustainability indicators providing pandemic resilience for residential buildings

**DOI:** 10.1016/j.mex.2021.101577

**Published:** 2021-11-09

**Authors:** Galym Tokazhanov, Aidana Tleuken, Mert Guney, Ali Turkyilmaz, Ferhat Karaca

**Affiliations:** Department of Civil and Environmental Engineering, The Environment & Resource Efficiency Cluster (EREC), School of Engineering and Digital Sciences, Nazarbayev University, Kabanbay Batyr Ave. 53, Nur-Sultan 010000, Kazakhstan

**Keywords:** Sustainability, COVID-19, Residential buildings, Green building

## Abstract

The method presented in this paper aims to support the sustainability assessment methods of residential buildings under pandemic conditions. The main purpose of the study is to review existing criteria of the well-known assessment tools and then to suggest a set of assessment measures for the emerging pandemic-resilient indicators. Current sustainability assessment methodologies mostly focus on the conventional sustainability pillars (Environmental, Social, Economic), whereas the proposed emerging sustainability assessment indicators include changes in sustainability requirements brought by the current pandemic. Firstly, a set of indicators with possible measures was identified; then, we reviewed several existing green building certification systems to identify their gaps and developed a foundation for each indicator. Finally, several round table discussions involving various stakeholders (e.g., engineers, designers, health care experts, academics) were conducted to consolidate the identified measures. The findings of the present study indicate that certain pandemic-resilient indicators are not fully addressed by existing assessment tools, pointing out the importance of the development of new measures to make them more suitable to use under pandemic conditions. Thus, the present study contributes to the building assessment methods by proposing a set of emerging assessment indicators with measures, which can be used by various professionals that would contribute to more sustainable buildings in upcoming pandemics.

• A 5-point scale was used to assess the indicators, and various stakeholders in a previous study identified their weights.
• The methodology introduces new pandemic-related indicators into the conventional sustainability concept.
• The assessment measures are rapid and economically efficient to apply for any residential building.

## Graphical abstract

**Figure:**
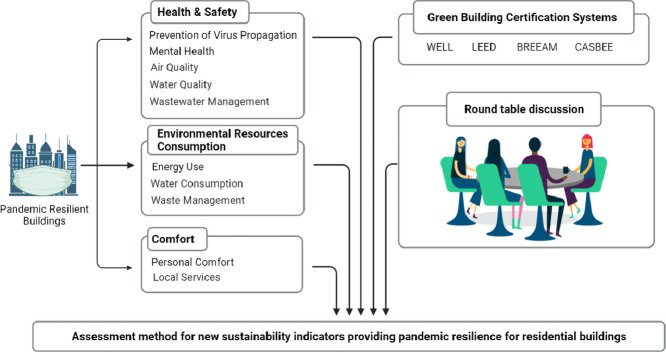

Specifications table

**Table:** 

Subject Area	Environmental Science
More specific subject area	*Building Sustainability*
Method name	Pandemic Resilience Assessment Method
Name and reference of original method	*Not applicable*
Resource availability	*Not applicable*

## Method details

COVID-19 pandemic has affected the world by causing various changes in people's daily lives. To prevent the rapid propagation of coronavirus, strict quarantines have been introduced globally to improve social distancing. Thus, residential buildings have changed their functionality from shelters to working, studying, and leisure places. This shift towards the increased importance of residential buildings, in turn, raises attention to their sustainability, too. Therefore, green building certification systems (GBCSs) might require technical improvements for better adaptation to new pandemic realities, considering that the world is predicted to meet more zoonotic disease outbreaks in the future [1]. Thus, the general aim of this research project is to develop such residential buildings’ criteria that would take into account occupants’ needs during pandemics, too. In the authors’ previous research, first, a literature review of the state-of-the-art articles, news, and blogposts on COVID-19 effect on residences was conducted [2]. It was followed by a development of indicators for pandemic-resilient residences, their hierarchical categorization into sub-categories and categories, and a comprehensive comparison of the pandemic-resilient indicators with sustainability indicators of existing GBCSs (The WELL Building Standard (WELL) [3], Leadership in Energy and Environmental Design (LEED) [4], Building Research Establishment Environmental Assessment Method (BREEAM) [5], Comprehensive Assessment System for Built Environment Efficiency (CASBEE) [6,7]. These indicators (or criteria) are mainly related to environmental and social pillars of sustainability and give particular focus to the health and safety of the building's residents. Next, a comprehensive discussion involving various experts, which included round tables and surveying activities with stakeholders from different disciplines (academy, medicine, and industry) were conducted with further analysis of their opinions towards new sustainability indicators towards pandemic resilience [8].

The present paper describes the methodology which identifies emerging measures and allocates scores to those pandemic-resilient criteria. All criteria have been scored quantitatively on a 5-point scale. While presenting the suggested scoring, the study also compares the selected existing GBCSs for the availability of similar criteria and their corresponding scoring. Several popular GBCSs (WELL, LEED, BREEAM, CASBEE) for comparison were chosen based on the number of citations in research articles, their extensive industrial use, and their focus on sustainability and residents’ wellness [7,9]. The main focus in assigning scores for the assessment criteria was to identify which practices would have a higher impact on sustainability during a pandemic (specifically based on the experience of the COVID-19 pandemic). Although the indicators mainly focus on multi-residential buildings, they do not belong to a city-specific context. The assessment is possible and suggested to be completed by a wide range of users (e.g., designers, architects, engineers, and residents). It provides an extensive set of building criteria aiming to provide residents with health, safety, comfort, and sustainable use of building services.

## Category 1: health & safety (H&S)

This category covers various main aspects of residential buildings that relate to the health & safety of their residents. Lockdowns during pandemics have made everyone experience isolation in their homes and highlighted the significance of maintaining a safe and healthy environment within residential buildings.

### Sub-category: PVP (Prevention of virus propagation)

The main goal of a lockdown is to decrease the rate of the virus spread (aiming at “flattening the curve”) as well as the number of patients during pandemic peaks. Hence, it is of utmost importance to decrease the virus propagation using all possible ways. Table 1 summarizes the existing assessment weights suggested in the reviewed GBSCs, and the assigned points.

**Table 1:** Assessment weights of existing GBCSs for PVP indicators.

*Indicator*	*WELL*	*LEED*	*BREEAM*	*CASBEE*
PVP1: Use of new smart/innovative technologies	1 point / 77 points		7 points (7% of total)	
PVP2: Use of touchless technologies				
PVP3: Self-cleaning spaces	1 point / 77 points	1 point / 110 points		
PVP4: Proper selection of indoor materials	1 point / 77 points	1 point / 110 points	3 points (1.35% of total)	5 points (12.75% of total)
PVP5: Natural light	1 point / 77 points		3 points (1.8% of total)	3 points (5% of total)
PVP6: Adjustability of indoor temperature and humidity	2 point / 77 points	3 point / 110 points		3 points (4.35% of total)

*PVP1: Use of new smart/innovative technologies.* Smart and innovative technologies, both in households and buildings, can efficiently prevent virus propagation [10]. The current indicator addresses the application of technologies in order to hinder the virus spread. WELL suggests using shoe cleaning systems, rollout mats, and air to be slowed down via one of the following mechanical ventilation systems: two entry doors, revolving entry doors, three or more doors separating the occupied space and outside [3]. BREEAM suggests giving one point for each applied BRE Global approved innovation [5]. The proposed method offers to award one point for each smart/innovative technology aimed to prevent virus propagation in the building (up to 5 points):

• Revolving or secured gates entrance doors forcing people to enter individually.
• At least three normally-shut doors that separate occupied space from the outdoors (e.g., apartment-building and building doors entrance, and having a building entry vestibule with two normally-closed doorways).
• Enhanced ventilation to maintain the freshness of the air.
• Use of thermo-visors to measure temperature and notify residents (AI face identification), if needed.
• Use tracking technology to identify if potentially infected people are around.

The criteria listed above are mainly based on the WELL method and suggestions during the round table discussion with experts.

*PVP2: Use of touchless technologies.* Another main route of virus propagation is via contact with infected surfaces. Thus, the avoidance of contact with potentially infected surfaces can decrease the chances of getting infected. Current technological progress allows us to use various devices to avoid unnecessary contact with household surfaces and surfaces within a building (i.e., elevator buttons, apartment doorknobs) [11], [12], [13]. Hence, it is critical to use technological development to decrease the spread of a virus by decreasing the number of contacts with surfaces. No reviewed GBCSs are addressing the indicator, and the proposed method suggests giving one point for each touchless technology (motion sensor, keycard swiping, pressure sensors) that prevents the contact of hands with potentially infected surfaces both within the apartment (touchless household technologies) and within the building (apartment doors, elevator doors, entry doors) (total of maximum 5 points).

*PVP3: Self-cleaning spaces.* During the pandemic, the people responsible for cleaning the streets and public spaces were among the populations most vulnerable to infections. It could be observed those people wearing protective clothing and equipment during the procedures of disinfecting and cleaning. It was also experienced that regular cleaning of surfaces is of utmost importance to fight a pandemic. Implementing self-cleaning spaces might avoid the risk of getting infected from high-touch surfaces. WELL [3] addresses the indicator suggesting a plan for cleaning procedure including:

• Surface classification.
• Control systems.
• Training for the maintenance.
• Cleaning frequency.
• Specific needs depending on the cleaning location (entrance, crowded area, etc.).
• Management of waste.

LEED methodology [4] suggests the use of disinfectants listed in EPA's List N: Disinfectants for use against SARS-CoV-2. The proposed method adapts and combines the reviewed methodologies, and points are distributed as follows:

• Used disinfectants properly disposed to avoid possible contact with humans (1 point).
• Identification of high-touch surfaces and frequently occupied spaces and having a cleaning plan (2 points).
• Regular use of EPA List N certified disinfectants (2 points).

The criteria selected is a combination of LEED and WELL methodologies’ parts, which suit the indicator's aim the best.

*PVP4: Proper selection of indoor materials.* Viruses can be active for longer or shorter timespans on surfaces, depending on their type and characteristics. For example, copper is a metal that kills pathogens [14,15]. Moreover, the porous structure of some materials, such as cardboard, may trap viruses in its porous surface structure and thus prevent its further transmission [15]. All four GBCSs [3], [4], [5], [6] cover this indicator, mainly suggesting the use of certified materials from officially confirmed lists of materials. WELL covers the indicator more broadly compared to other GBCSs: high-touch surfaces, including doorknobs, handles, elevator buttons, and light switches, are covered with an anti-microbial material, which is abrasion-resistant, non-leaching, and meets EPA requirements [3].

The proposed method distributes points as follows:

• Selection of materials that are in a list of anti-microbial materials verified by a qualified agency (1 point).
• Create a list of surfaces based on the frequency of touch (1 point).
• Percentage of surfaces from the list covered with anti-viral material:
  • 40–60%: 1 point.
  • 61–80%: 2 points.
  • 81–100%: 3 points.

All four GBCSs contributed to the criteria development of this indicator.

*PVP5: Natural light.* The sun radiation could kill pathogens and thus deactivate them [16,17]. Even though the sunlight is not effective against all pathogens [18], it nevertheless can create a healthier environment in a home. Moreover, natural light is directly related to the mental health of the residents, who are under stress during a lockdown. WELL awards points based on window size, visible transmittance, and uniform color [3]. BREEAM suggests conducting a simulation of microclimate or solar exposure study [5]. CASBEE uses the calculation of daylight factor as criteria to address this indicator [6]. The proposed method suggests to give points based on the direct sunlight area of the room:

• 0–20% (1 point).
• 21–40% (2 points).
• 41–60% (3 points).
• 61–80% (4 points).
• 81–100% (5 points).

Since the aim of the indicator is to reduce the pathogen level via natural light, the criteria of existing GBCSs are not suitable. Hence, the proposed criteria are based on the round table discussion with experts.

*PVP6: Adjustability of indoor temperature and humidity.* The temperature and the humidity of air affect the transmission efficiency of viruses [18,19], where high humidity and high temperature might reduce the ability of a virus to spread. Hence, the airborne transmission pathway within a building or an apartment can be limited via an effective control of those parameters. All four reviewed GBCSs suggest methodologies to assess the indicator. The criteria include controlling humidity and temperature for conditions suitable for the pre-pandemic situation in a way more related to comfort. WELL awarded points considering the following characteristics [3]:

• Humidity: 30–50%.
• At least 95% of all working hours.
• ASHRAE Standard 55–2013 for thermal comfort is met by the use of hydronic radiant heating or an electric radiant system.

LEED's criteria include at least two conditioning zones and room-by-room control of temperature [4]. BREEAM suggests minimizing adverse conditions, creating a favorable microclimate, and public space optimization to sustain microclimate conditions as criteria for this indicator [5]. At the same time, CASBEE gives points based on the minimization of vertical temperature and humidity difference and temperature and humidity adjustment precision. The aim of this indicator differs from the existing GBCSs indicators, which are focused on comfort. Hence, existing GBCSs criteria were not used for the development of the proposed criteria.

The proposed methodology also suggests temperature and humidity adjustability to prevent the virus spread:

• Automatic control of temperature via AC or control valves on radiators (1 point).
• Separate control of air temperature room by room (2 points).
• Full range humidity control of the entire building (2 points).

This indicator aims to reduce the virus transmission efficiency.

### Sub-category: MH (Mental health)

The quarantine experience showed that long-time isolation could negatively impact personal mental health [2]. Under such conditions, mental health can be maintained reasonably in different ways, including keeping people busy with greenery, socializing within social distancing rules, and performing indoor physical activities. Table 2 shows weights and points of MH indicators addressed by existing GBCSs.

**Table 2:** Assessment weights of existing GBCSs for MH indicators.

*Indicator*	*WELL*	*LEED*	*BREEAM*	*CASBEE*
*MH1: Availability of greenery and gardens*	1 point / 77 points		4 points (2.8% of total)	5 points (9% of total)
*MH2: Availability of outdoor spaces in the building*				
*MH3: Access to common building spaces with sufficient safety and social distance*			4 points (1.8% of total)	5 points (2.25% of total)
*MH4: Household-level activity/sports spaces*	2 point / 77 points		4 points (1.8% of total)	

*MH1: Availability of greenery and gardens.* The presence of plants or other livings increases the mental state of a person [20]. The indicator addresses the availability of such greeneries that help residents maintain a healthy mental state during and after a pandemic. The WELL criteria include at least 25% of the outdoor area is landscape ground or gardens, plants on walls and potted plants indoors, 1% of the floor area must be covered for potted plants, 2% for wall plant, and water feature with proper sanitation [3]. BREEAM suggests consultation (quantity, location) with stakeholders regarding the green spaces with proper maintenance, and location should be within walking distance [5]. CASBEE gives points based on the efforts in greening the area, monitoring, and improving the contact between residents and flora and fauna [6]. The proposed method is mainly based on WELL criteria, which matches the aims of this indicator. The criteria are as follows:

• At least 25% of the outdoor area landscape ground or gardens (1 point).
• Availability of green space(s) within walking distance (1 point).
• Potted and wall plants and water (e.g., fountains) (1 point):
  • Potted plants cover 1% of the floor area.
  • Area of the wall plants are larger than 2% of the floor area.
  • One water feature for a building block or larger features for the building complex.
  • Proper sanitation (e.g., ultraviolet) of the water feature(s).
• Small gardens on the balconies (1 point).
• Indoor gardening for winter or total isolation times (1 point).

*MH2: Availability of outdoor spaces in the building.* Outdoor spaces are essential for residents’ mental health, especially during a quarantine, as their availability is critical to avoid depressed mood and mental stress [21]. No GBCSs cover such an indicator; the proposed method was suggested based on the experiences of the stakeholders:

• Availability of a private balcony or terrace (1 point).
• Availability of roof spaces (e.g., green roof) for residents’ use (2 points).
• Availability of other open spaces in the building within social distancing rules (2 points).

*MH3: Access to common building spaces with sufficient safety and social distance.* Socializing is one of the fundamental human needs. Hence, during a lockdown, there is a need for common spaces allowing people to socialize without the risk of getting infected. BREEAM gives points for inclusive design considering the public realm and open space [5]. CASBEE addresses some measures closely related to the aims of the indicator, which gives points based on the design of semi-outdoor and intermediate spaces, and psychologically rich zones and spaces for sentimental expression [6]. Similarly, the proposed criteria include the availability of common (semi-outdoor, intermediate) space for socializing (2.5 points) and adding a social distancing rule (2.5 points) as a measure.

*MH4: Household-level activity/sports spaces.* Physical activity is beneficial for the general health condition of an individual [22]. A lockdown hinders the ability to perform physical activity as it is not possible to visit sports centers or complexes nor to go outside for exercise. Hence, a space for sports activities is required to maintain a healthy lifestyle, which would also benefit the mental health of the residents. WELL covers this wider than BREEAM [5] (points for the availability of sport and recreation spaces), while the other two GBCSs don't address the indicator. The WELL criteria include the availability of a park with workout stations or free access to a gym with cardiorespiratory and muscle-strengthening training equipment within 0.8 km [3], and they were used as a basis for the proposed method, with additions of pandemic requirements:

• Availability of a public park with workout stations or free access to a gym with cardiorespiratory and muscle-strengthening training equipment (2.5 points).
• Keeping the social distances (e.g., open-air conditions, perfect ventilation, routine cleaning practices, limited access with age category, PCR testing or/and vaccination, schedule, and reservation-based entry). Instructions for safe use is provided (2.5 points).

### Sub-category: AQ (Air quality)

The main transmission route of the SARS-CoV-2 is via droplets carried via the air. Hence, maintaining clean air is essential to decrease getting infected and to maintain a healthy environment. Table 3 shows how existing GBCSs addressed AQ indicators.

**Table 3:** Assessment weights of existing GBCSs for AQ indicators.

*Indicator*	*WELL*	*LEED*	*BREEAM*	*CASBEE*
*AQ1: Efficiency of air filtration systems for pathogen propagation*	0.6 point / 77 points	Prerequisite		
*AQ2: Monitor and control indoor air pollution*	1 point / 77 points	1 point / 110 points	1 point (0.6% of total)	5 points (7.25% of total)
*AQ3: Control the airflows in micro spaces*	1 point / 77 points	3 point / 110 points	3 points (1.8% of total)	
*AQ4: Level of natural ventilation*	1 point / 77 points	1 point / 110 points	Mentioned as suggestion	5 points (5% of total)

*AQ1: Efficiency of air filtration systems for pathogen propagation.* Proper air filtration techniques should eliminate viruses that could suspend and travel in the air. Only WELL and LEED suggests such measures. It should be noted that LEED considers the indicator as a prerequisite and does not give any points for it. WELL include the use of UV germicidal irradiation or photocatalytic oxidation treatment technologies for spaces with more than ten people and availability of air quality maintenance and control [3]. LEED requires the use of filters with a minimum efficiency value of 8 [4]. The proposed criteria are mainly based on the WELL method and are as follows:

• High-efficiency particulate air (HEPA) filters for minimization of pathogens (2 points).
• One of the following to be used for the treatment of recirculated air, either as part of the ventilation system or as a separate device (2 points):
  a Ultraviolet germicidal irradiation.
  b Photocatalytic oxidation.
  c or other relevant/approved technologies.
• Having and recording routine maintenance (filter and sanitizer) (1 point).

*AQ2: Monitor and control indoor air pollution.* Air quality is difficult to assess without proper equipment. Hence, constant monitoring and control of air conditions are essential to control air pollution and increase air quality. The WELL criteria better fit the indicator's scope in its detail [3] than other GBCSs:

• Hourly monitoring of at least two of the following:
  • Particle count or mass.
  • CO_2_ concentration.
  • Ozone.
• Real-time monitoring of:
  • Temperature.
  • Humidity.
  • CO_2_ concentration.
• For 46.5 m^2^ or larger spaces: A demand-controlled ventilation system to maintain the concentration of CO_2_ below 800 ppm

LEED only mentions the prohibition of indoor tobacco smoking [2]. BREEAM gives points for microclimate simulation with air direction and speed [5]. CASBEE requires constant monitoring of CO_2_, NO_x_, SO_x_ [6].

The proposed method adopts the most appropriate measures available in the GBCS as:

• Monitoring of two of the following parameters with an interval of once in an hour (2 points):
  • PM.
  • Ozone.
  • CO_2_.
  • Temperature.
  • Humidity.
• If all demand-controlled ventilation systems for spaces are monitored based on limit values (2 points).
• Recording of the parameters kept for a minimum of one year (1 point).

*AQ3: Control the airflows in micro spaces.* Air circulation can transport viruses from one place to another. If there is an infected person in the house, it is crucial to provide proper isolation, including airflow. The circulation and flow of air masses should be carefully controlled to avoid spreading the virus. The GBCSs are addressing such measures except for CABSEE. WELL suggest using self-closing doors and expelling air rather than recirculating it [3]. LEED requires sealing of wall, floor, and ceiling penetrations, weather-stripping all doors, blower door test for sealing check [4]. BREEAM points are given for maintaining favorable microclimatic conditions via factors including air movement, direction, and speed [5]. However, their intention was not to stop the virus from spreading but mainly to ensure better insulation between micro spaces. Preventing the spreading of viruses follows the same logic as the existing measures suggested by the others, so the present method adapts these measures:

• Control of the air movement by negative pressure and self-closing doors (1 point).
• Avoid recirculation of the air via air exhaustion (1 point).
• Room Ventilation systems with variable speeds in different rooms or, at least, one in one room (3 points).

*AQ4: Level of natural ventilation.* Natural ventilation is an environmentally friendly and effective way to refreshen indoor air. Ventilation is essential during a pandemic to increase air freshness [23,24]. WELL has two main criteria: (1) a window can provide ventilation enough to keep the CO_2_ level below 800 ppm, (2) outdoor temperature, ozone, PM_10_, and humidity levels monitored within 1.6 km of the building with residents’ notification when conditions are unacceptable [3]. LEED requires natural ventilation if the building was occupied for at least 1 h and use of MERV 13 or above filters [4]. BREEAM only suggests the reduction of energy via natural ventilation [5]. CASBEE criteria include the availability of certain openable windowed [6]. WELL partially adapted for this indicator's assessment. The proposed criteria are:

• All the rooms have an openable window for natural ventilation (2 points).
• Outdoor air monitoring stations located within 1.6 km proximity provides the residents live data with alert notices (e.g., via smartphones) in case of unsafe environmental conditions (2 points).
• Adjustable openings and types of windows for control of natural ventilation (1 point).

### Sub-category: WQ (Water quality)

*WQ1: Safety measures of drinking water and/or tap water from contamination.* Tap water is not potable in many cities. During a quarantine, people may not be able to go outside and gain access to potable water. It is essential that the building has access to fresh drinking water clean from any pathogens. Only WELL and BREEAM address the indicator (Table 4), where BREEAM is more related to eliminating contamination focusing on drainage plan, treatment of run-off water, use of shut-off valves, and oil separators [5]. WELL mainly addresses monitoring turbidity, total coliforms, lead, arsenic, mercury, and copper with data recording [3]. The proposed method merges both monitoring and prevention of contamination under pandemic conditions:

• Use high-performing ultrafiltration or nanomembrane filters for the cleaning of drinking water (2 points).
• Provision of tab water with no turbidity, UV irradiation, and appropriately dosed free chlorine (2 points).
• Regular testing and monitoring for pathogens during pandemic times (1 point).

**Table 4:** Assessment weights of existing GBCSs for WQ indicators.

*Indicator*	*WELL*	*LEED*	*BREEAM*	*CASBEE*
*WQ1: Safety measures of drinking water and/or tap water from contamination*	2 point / 77 points		3 point (1% of total)	
*WQ2: Maintenance and/or decontamination of the building water system for infection*	1 point / 77 points	1 point / 110 points		

*WQ2: Maintenance and/or decontamination of the building water system for infection.* UNICEF and WHO [16] recommend treating water systems against pathogens at the building level via filtration, disinfection, and chlorination. LEED generally mentions maintaining water quality and does not give specific criteria for this indicator [4]. The same BREEAM indicator that addresses WQ1 also covers WQ2. WELL was mainly used as a basis for the development of the proposed method, which has criteria related to the removal of organic chemicals, sediments, microbes, and maintenance and control of water quality [3]. The proposed method includes the following criteria:

• Filtration of water via activated carbon filters (1 point).
• Filters to remove sediments with a pore size of 1.5 µm or less (1 point).
• Elimination of microbes via UV germicidal irradiation, NSF rated filters (1 point).
• Chlorination or ozonation of water (1 point).
• Recording of maintenance and water quality (1 point).

### Sub-category: wwm (Wastewater management)

*WWM1: Specific measures to limit virus propagation at the household level.* Viruses can be found in wastewater and can become airborne by aerosolization [25]. Hence, water pipes, sinks, and other water manipulation systems should be appropriately sealed. No reviewed GBCS covers the indicator, and the proposed method offers measures based on the current state of technologies related to the goals of the indicator:

• Ensure proper sealing of pipes, sinks, water cooling systems. (e.g., sensing systems to check the piping leakage per apartment) (2 points).
• Maintain negative pressure in wastewater pipelines (1 point).
• If greywater is separately collected, it should be reused in those cases which have limited human contact, ensure pre-treatment of water (1 point).
• Separating wastewater system between possibly infected flats and zones in the building system (e.g., having different water collection systems for intensive care units or toilets for sick people) (1 point).

*WWM2. Availability of separate toilets for infected.* Separate toilets for infected people is a potential solution to maintain healthy conditions of living. A shared toilet increases the chances of getting infected [26]. Hence, it is a good practice to separate the toilet for infected people. No GBCS is covering the indicator, and the proposed method suggests new measures:

• Separate toilets in every apartment for infected people (not suitable for temporary situations) (3 points)
• Self-disinfection of the toilet space (2 points).

*WWM3. Separation of greywater.* Greywater has a higher risk of transmitting the virus due to the lower level of treatment it goes through. Hence, separating greywater from other water sources is essential to provide a clean piping system [27]. No GBCS is covering the indicator. The proposed criteria are as follows:

• Separate pipes and disposal pathways for greywater (2 points).
• Disinfection of greywater (2 points).
• If greywater is disposed to a soak-away pit, it should be fenced off within the residential facility grounds to prevent tampering and to avoid possible exposure in the case of overflow (1 point).

## Category 2: environmental resources consumption (ERC)

### Sub-category: EU (energy use)

The global consumption of energy via household activities has increased due to lockdown measures forcing people to stay at their homes [28]. The rapid increase in energy use might be caused by the wide use of different systems and devices such as television, AC, ICTs (Information and Communication Technologies), cooking, and laundry. Table 5 shows points and weights of EU indicators by existing GBCSs.

**Table 5:** Assessment weights of existing GBCSs for EU indicators.

*Indicator*	*WELL*	*LEED*	*BREEAM*	*CASBEE*
*EU1: Access to backup energy sources*				
*EU2: Promotion of sustainable and alternative energy sources*		4 points / 110 points	11 point (4.1% of total)	5 points (2% of total)
*EU3: Use of energy-efficient appliances*		51 points / 110 points	6 points (4.1% of total)	5 points (6% of total)

*EU1: Access to backup energy sources.* Increased and ongoing energy consumption of residential buildings might cause a failure of energy supply. For a comfortable stay in the house, a stable and continuous energy supply is a critical factor. Hence, there is a need to develop a backup energy source to supply the residential building during an emergency. No GBCS is covering the indicator. The proposed criteria are:

• Presence of backup energy source for emergency (3 points).
• Can supply the whole building for a minimum of an hour (1 point).
• Can supply the building for 24 or more hours (1 point).

*EU2: Promotion of sustainable and alternative energy sources.* Increased consumption of non-renewable energy leads to issues related to the environment and sustainability. Thus, sustainable renewable energy sources should be used as a constant energy supply for buildings. All reviewed GBCSs except WELL address the indicator (Table 5). LEED suggests giving one point for every 500 kWh/y produced by renewable electricity generation [4]. CASBEE gives points based on the amount of natural energy usage from renewable sources, natural light usage, natural ventilation, and geothermal energy [6]. BREEAM using a percentage of carbon reduction [5] has similarities with the proposed method:

Sustainable energy covers:

• 0–25% of the total consumed energy (2 points).
• 26–50% of the total consumed energy (3 points).
• 51–75% of the total consumed energy (4 points).
• 76–100% of the total consumed energy (5 points).

*EU3: Use of energy-efficient appliances.* Using energy-efficient technologies and appliances will decrease the energy consumed by household activities. Three GBCSs cover the indicator (Table 5). LEED [4] gives points for:

• Annual energy use.

Points based on a percentage of energy use reduction (90% reduction - 30 points maximum)

• The energy factor of the storage water heater is 0.59 for gas and 0.92 for electricity.
• High-efficiency, ENERGY STAR-qualified appliances (e.g., refrigerator, ceiling fans, and dishwasher).
• High-efficiency lighting.
• HVAC equipment, which is more efficient than ENERGY STAR for Homes, version 3.
• ENERGY STAR-qualified domestic hot water equipment.
• Efficient hot water distribution system based on pipe length or pipe volume.
• HVAC systems are commissioned by a specialist with North American Technician Excellence certification.
• Minimize air leakage.

BREEAM gives points based on energy savings via demand and fabric efficiency [5], and CASBEE assigns points based on “energy reduction ratio = energy saved/standard energy consumption” [6]. CASBEE methodology was found to be the most suitable to assess this indicator. Hence, the proposed criteria are:

• Energy reduction ratio < 0.25 (2 points).
• 0.26 ≤ Energy reduction ratio < 0.5 (1 point).
• 0.51 ≤ Energy reduction ratio < 0.75 (1 point).
• 0.76 ≤ Energy reduction ratio (1 point).

Energy reduction ratio = energy saved/standard energy consumption.

### Sub-category: WM (waste management)

*WM1: Proper segregation of medical waste.* For safety measures, personal protective equipment, including gloves and masks, are used extensively during pandemics. Proper management of the waste that is potentially infected is needed and requires special attention. [29]. Medical waste can be separated and processed separately with sufficient safety measures. No GBCS is addressing the indicator, and the proposed criteria are as follows:

• Separate collection of medical waste (3 points).
• Safe management of medical waste (2 points).

*WM2: Disinfection of household waste.* Household waste generated by infected people can contribute to the spread of the virus. Hence, it is essential to conduct proper disinfection of the waste to prevent virus propagation. No GBCS is addressing the indicator, and the proposed measure is to give 5 points for additional safety measures on disinfecting the household waste before it is in contact with people.

*WM3: Management of an increased amount of waste.* The coronavirus pandemic has resulted globally in an increased amount of waste due to reasons such as people using protective equipment, e.g., masks and gloves, and constantly changing them [30]. Another example is the increased use of delivery services that utilize single-use packaging generating large quantities of additional waste [31]. No GBCS is addressing the indicator, and the proposed measure is to give 5 points for additional waste management measures for residents to use recyclable materials and use fewer single-use packages during emergency times.

### Sub-category: WC (water consumption)

Household water consumption has increased during a pandemic due to several reasons, including increased handwashing, higher frequency of laundry, household disinfection activities, and home cooking [32]. The new conditions raise a need to develop stable access to water sources and their effective use.

*WC1: Access to alternative water sources.* During a case of increased water consumption, it is essential to ensure a constant water supply, which requires alternative water sources in emergencies. All GBCSs except WELL address the indicator (Table 6). LEED gives points based on reduction of landscape water consumption via smart scheduling technologies, rainwater capture, reclaimed water, on-site treatment of water [4]. BREEAM focuses on the percentage of hard surfaces used for rainwater collection [5]. CASBEE criteria include water-saving equipment, temporary storage of wastewater, use of well water, gray water, and rainwater, and rainwater storage tank [6]. A combination of all reviewed methodologies was used to develop the proposed method:

• Existence of rainwater storage tank for technical water use (2 points).
• Filters to convert rainwater into potable water (2 points).
• Storage tank for greywater and its filtration for technical use (1 point).

**Table 6:** Assessment weights of existing GBCSs for WC indicators.

*Indicator*	*WELL*	*LEED*	*BREEAM*	*CASBEE*
*WC1: Access to alternative water sources*		12 points / 110 points	3 points (1% of total)	5 points (1.55% of total)
*WC2: Use of water-efficient appliances and fixture*		6 points / 110 points		5 points (1.1% of total)

*WC2: Use of water-efficient appliances and fixtures.* The application of water-saving efficient equipment within a household can decrease the amount of used water. LEED and CASBEE are addressing the indicator (Table 6). However, no GBCSs suggested the easy way to measure the water savings. Hence, the proposed method was developed independently from the reviewed methods:

• Water usage reduction ratio < 0.25 (2 points).
• 0.26 ≤ Water usage reduction ratio < 0.5 (3 points).
• 0.51 ≤ Water usage reduction ratio < 0.75 (4 points).
• 0.76 ≤ Water usage reduction ratio (5 points).

Water usage reduction ratio = saved water/total consumed water.

## Category 3: comfort

### Sub-category: PC (Personal comfort)

*PC1: Specific emphasis on household-level ICT infrastructure access.* During the pandemic, most businesses and services could be forced to switch to online systems. Hence, the use of the internet (including online studying, working, medical consultation, food ordering) has rapidly increased [33,34]. Only CASBEE addresses the indicator (Table 7), giving points for the usage of optical fiber cable, metal cable, presence of cellular telephone network, personal handy phone network, and at least two communication links [6]. Since the indicator aims to ensure stable ICT service, the proposed criteria include giving 5 points for more than one reliable and constant ICT connection (e.g., optical fiber cable, metal cable, cellular telephone network, personal handy phone network).

**Table 7:** Assessment weights of existing GBCSs for PC indicators.

*Indicator*	*WELL*	*LEED*	*BREEAM*	*CASBEE*
*PC1: Specific emphasis on household-level ICT infrastructure access*				5 points (0.2% of total)
*PC2: Levels of indoor space adjustability*				5 points (1.55% of total)
*PC3: Personal space*			2 points (2.7% of total)	
*PC4: Design level adjustments on noise insulation and acoustics*	2 points / 77 points		3 points (1.8% of total)	5 points (2% of total)

*PC2: Levels of indoor space adjustability.* It is unavoidable to combine within the house borders various aspects of life during a quarantine, including work, sports, and leisure time. The adjustability of the space allows residents to transform the rooms quickly based on the occupants’ needs. In addition, work organization can be increased via home adjustability technologies [35]. CASBEE is the only GBCS that covers such measures (Table 7), giving layout adaptability points without any quantitative measure [6]. Proposed criteria are based on round table discussion and give points based on adjustable area:

• Adjustable area ratio up to 0.10 (2 points).
• Adjustable area ratio up to 0.20 (3 points).
• Adjustable area ratio up to 0.30 (4 points).
• Adjustable area ratio up to 0.40 (5 points).

Adjustable area ratio = adjustable area/total area.

*PC3: Personal space.* The coronavirus pandemic showed that personal space is a critical factor for the quality of social life for a person. Families without sufficient space for family members may face difficulties in establishing the boundaries of private space. Availability of private space for work, study, or other personal reasons is essential to maintain a healthy mental state and low-stress levels [36]. Only BREEAM addresses the indicator, albeit without detailed criteria for assessment (Table 7). BREEAM gives points if the developer commits to provide minimum best practice standards of space [5]. The proposed criteria are the result of the round table discussions and are as follows:

• Room to member ratio < 0.25 (no points).
• 0.26 ≤ Room to member ratio < 0.5 (1 point).
• 0.51 ≤ Room to member ratio < 0.75 (2 points).
• 0.76 ≤ Room to member ratio < 1 (3 points).
• 1 = Room to member ratio (5 points).

Room to member ratio = number of rooms/family members

*PC4: Design level adjustments on noise insulation and acoustics.* A low level of noise pollution is essential for the comfortable stay of the residents. Some research results show that acoustic comfort is more important than thermal, light, or air conditions [37]. All reviewed GBCSs except LEED are addressing the indicator (Table 7). WELL suggests giving points for reduction of sound via proper sealing, staggering of gypsum board, packing of wall penetrations. The acoustic plan also should consider quiet and loud zones and equipment generation noises [3]. BREEAM suggests conducting noise impact assessment, and noise level not greater than +5 dB (day), +3 dB (night) compared to background [5]. CASBEE assigns credits for the use of low-noise equipment [6]. BREEAM suggests the most suitable criteria, the primary basis for suggesting the current method: 5 points are given for noise level difference less than +5 dB (day), +3 dB (night) compared to the background noise.

### Sub-category: LC (local services)

*LS1: Availability of self-dependent services in the residential complexes.* Local services, especially food and medical supply, are essential for the sustainable functioning of a district or area when the transportation and movement of goods are limited during a quarantine. Local food shortage was experienced during the quarantines when whole residential complexes were closed [38]. The limitation or complete blockage of inter-city transportation, including goods, showed the interdependence of smaller city regions. Hence, there is a need to create an independent and sustainable supply of food at the local (or building) level, which will also prevent the spread of the virus [39]. The indicator is addressed only by BREEAM, which suggests an economic study involving consultation with stakeholders (Table 8). BREEAM criteria include the economic study, including neighborhood development, local strategic master plan to be carried as a review of the current demographic profiles and future of the local area. The development plan includes consultation with the community and appropriate stakeholders [5]. Since no GBCSs proposed suitable criteria for this indicator, the proposed method's points are given based on the provision of local (availability in the residential complex or campus area, or having a reliable online delivery option) services listed:

• Ratio of local services < 0.25 (1 point).
• 0.26 <= Ratio of local services < 0.5 (2 points).
• 0.51 < = Ratio of local services < 0.75 (3 points).
• 0.76 <= Ratio of local services < 1 (5 points).

**Table 8:** Assessment weights of existing GBCSs for LC indicators.

*Indicator*	*WELL*	*LEED*	*BREEAM*	*CASBEE*
*LS1: Availability of self-dependent services in the residential complexes*			1 point (2.7% of total)	
*LS2: Urban/community farming*	1 point / 77 points		1 point (2.7% of total)	

Ratio of local services = number of local services/ number of total services.

*LS2: Urban/community farming.* The sustainability and independence of the local food supply can be increased via the development of urban farming [40], which can help maintain sufficient food reserves when no external interference is allowed due to quarantine measures. It can be accomplished on several levels, including growing vegetation within the apartment, building, or even at a community level. In addition, farming at any level could facilitate the healthy mental condition of the residents [41]. WELL and BREEAM address the indicator (Table 8). WELL includes 0.1 m^2^ of gardening space per occupant within 0.8 km and provision of proper conditions for gardening, including medium, irrigation, lighting, plants, and tools [3]. BREEAM gives points for local needs identification, including the space for vegetable and fruit growing [5]. The WELL criteria were useful for the development of the proposed criteria, which are as follows:

• Apartment-level farming (1 point).
• Apartment and/or building level farming with 0.1 m^2^ of gardening space per resident (+2 points).
• Community-level farming with proper conditions created (+2 points).

## Conclusion

Since the start of the COVID-19 pandemic, people all over the globe have been experiencing the difficulties of lockdown measures confining them to their houses for long periods of time, indicating a need to change the design of our buildings to make them pandemic resilient. However, such changes require appropriate assessment tools to evaluate the sustainability of buildings under pandemic conditions accurately. The present study shows that existing green building certification systems do not satisfy such a need as they do not fully cover the much-needed pandemic-resilient indicators. The proposed measures in this study contribute to filling this gap regarding the development of pandemic-resilient residential buildings. The proposed method and these emerging criteria will be helpful for multiple stakeholders (e.g., engineers, architects, construction designers) to provide better and more sustainable building solutions during future pandemics.
